# Flexible and rapid construction of viral chimeras applied to hepatitis C virus

**DOI:** 10.1099/jgv.0.000530

**Published:** 2016-09

**Authors:** C. Patrick McClure, Richard A. Urbanowicz, Barnabas J. King, Sara Cano-Crespo, Alexander W. Tarr, Jonathan K. Ball

**Affiliations:** ^1^​ School of Life Sciences and NIHR Nottingham Digestive Diseases Biomedical Research Unit, The University of Nottingham, Nottingham University Hospitals NHS Trust, Nottingham, UK

**Keywords:** viral chimera, reverse genetics, hepatitis C virus, cell culture, DNA assembly, In-Fusion cloning

## Abstract

A novel and broadly applicable strategy combining site-directed mutagenesis and DNA assembly for constructing seamless viral chimeras is described using hepatitis C virus (HCV) as an exemplar. Full-length HCV genomic cloning cassettes, which contained flexibly situated restriction endonuclease sites, were prepared via a single, site-directed mutagenesis reaction and digested to receive PCR-amplified virus envelope genes by In-Fusion cloning. Using this method, we were able to construct gene-shuttle cassettes for generation of cell culture-infectious JFH-1-based chimeras containing genotype 1–3 E1E2 genes. Importantly, using this method we also show that E1E2 clones that were not able to support cell entry in the HCV pseudoparticle assay did confer entry when shuttled into the chimeric cell culture chimera system. This method can be easily applied to other genes of study and other viruses and, as such, will greatly simplify reverse genetics studies of variable viruses.

The genetic diversity of viruses often demands representation of many isolates in experimental systems in order to comprehensively phenotype an organism and to probe efficacy of prophylactic and therapeutic intervention (Burton *et al.*, 2012; Das *et al.*, 2013; deCamp *et al.*, 2014; Imhof & Simmonds, 2010, 2011; Pietschmann *et al.*, 2006). Reverse genetics systems developed to study host–pathogen interaction are typically constructed by PCR amplification and gene or genome cloning into plasmid vectors (Das *et al.*, 2013; deCamp *et al.*, 2014; Dutta *et al.*, 2013; Imhof & Simmonds, 2010, 2011; Lindenbach *et al.*, 2005; Pietschmann *et al.*, 2006; Urbanowicz *et al.*, 2015). This involves a protracted multistep process, utilizing iterative rounds of restriction endonuclease (RE) digestion, stitch/fusion PCR and subsequent enzymatic modifications (Edmonds *et al.*, 2010; Imhof & Simmonds, 2010; Lindenbach *et al.*, 2005; Steinmann *et al.*, 2013). Each amplification and ligation reaction can be error-prone, frequently requiring corrective back-mutation steps. Such workflows rely on either naturally occurring RE sites, which are often suboptimally located, or on the generation of novel RE sites, which can impact on important biological properties such as RNA structure (You *et al.*, 2004) even when mutations are synonymous. Reliance on RE-based cloning can also be adversely affected by the presence of internal RE sites in the target gene, due to natural genetic variation of the virus. These experimental difficulties have limited the range of reference phenotyping systems available, despite their crucial importance in vaccine and treatment development and monitoring (Burton *et al.*, 2012; deCamp *et al.*, 2014; Imhof & Simmonds, 2011).

Hepatitis C virus (HCV) displays extensive genetic heterogeneity, particularly in the genes encoding envelope glycoproteins (E1 and E2). Studies of the impact of natural variation on antiviral sensitivity (Imhof & Simmonds, 2010) or on neutralization sensitivity to mAbs (Ball *et al.*, 2014) or vaccine-sera (Chmielewska *et al.*, 2014), have suffered significantly from the drawbacks described above and the current lack of culture systems for wild-type, patient-derived isolates. This has resulted in the development of drug sensitivity phenotyping methods that require significant genetic manipulation (Imhof & Simmonds, 2010, 2011) or the use of very small panels of infectious virus that are potentially unrepresentative of patient-derived virus for antibody studies (Keck *et al.*, 2013).

DNA assembly technology (Irwin *et al.*, 2012) was therefore employed to develop a novel strategy for generating chimeric molecular cloning cassettes. Using this method, a panel of 49 functional full-length HCV genomes containing patient-derived E1E2 has been derived. This has revealed novel insights into their function that was hitherto impossible using existing phenotyping resources, such as pseudoparticle (pp) assays. Importantly, this approach can be widely used in reverse genetics studies of any genetically variable virus, or indeed other organisms, employing plasmid constructs.

Sequence files of parental HCV genotype 1 (Gt1) Bi-Gluc-H77C(1a)/JFH (T2700C, A4080T) (Reyes-del Valle *et al.*, 2012) and Gt2 J6/JFH-1 (Lindenbach *et al.*, 2005) chimeric clones were screened for RE sites using the online NEBcutter 2.0 tool [http://nc2.neb.com/NEBcutter2/, New England Biolabs (NEB)]. FseI was identified in both parental clones as a non-cutting RE site with 3′ overhangs, and therefore leaving the smallest footprint in standard In-Fusion cloning (Clontech Laboratories Inc), retaining only two bases (5′ GG, 3′ CC) of the RE site post-cloning at each chimeric junction. The desired chimeric junction points at the 5′ end of the signal peptide of E1 and the 3′ C terminus of E2, were scanned for the retained GG and CC dinucleotide motifs, respectively, and candidate sites located (Fig. 1a). In the absence of convenient dinucleotide motifs, enzymatic blunting of 5′ overhangs could be performed to extend choice of cloning sites to single bases. The reliance on the presence of only the double-stranded remnant at the 3′ overhanging RE site thus increases potential chimeric junction options by several orders of magnitude.

**Fig. 1.**
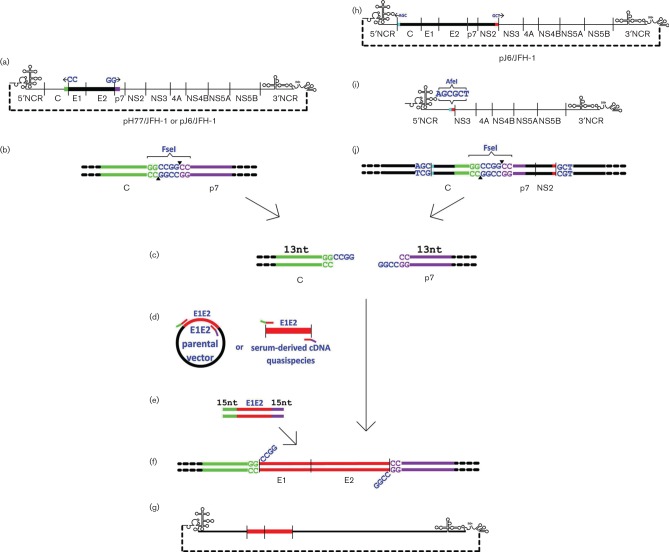
ΔE1E2FseI and ΔCore-NS2 AfeI HCV cassette and chimera construction schematic. The E1E2 region of the full genomic parental clone is deleted between the naturally occurring non-overhanging termini of an RE site not found in the parental clone [FseI here, codons 171 to 742 (H77) or 748 (J6)], and replaced with the complete novel RE site by a single SDM reaction (a). The ΔE1E2FseI plasmid cassette (b) is RE digested at the novel FseI RE site (c) and assembled by In-Fusion cloning (f) with patient-derived E1E2 (d) amplified with 15 bp terminal homology to the digested cassette (e). The resulting chimera contains only wild-type sequence from both parental full-length genome and patient-derived E1E2 isolates (g). Similarly, to generate intergenotypic cassettes the Core-NS2 region of the full genomic parental clone is deleted between the naturally occurring non-overhanging termini of an RE site absent in the parental clone (h, AfeI here, codons 2–1031), and replaced with the complete novel RE site by a single SDM reaction (i). The ΔCore-NS2 AfeI plasmid cassette is RE digested at the novel AfeI site and assembled by In-Fusion cloning with *in vitro* synthesized Core-NS2 with 15 bp terminal homology to the digested clone and E1E2 region replaced with a unique RE site (FseI here, pre-deleting codons 171–734). The resulting ΔE1E2FseI cassette (j) can then receive patient-derived E1E2 of the same genotype as the inserted core and NS2 genes to create functional intergenotypic chimeras (c–g).

Site-directed mutagenesis (SDM) primers were then designed using the online NEB BaseChanger tool (http://nebasechanger.neb.com/) to simultaneously knockout the parental E1E2 sequence between the GG and CC junction points and introduce the 4 bp motif CCGG to create a complete FseI RE site (GGCCGGCC, Fig. 1b; primers 1, 2, 14 and 15, Table S1, available in the online Supplementary Material). All primers were synthesized by Eurofins. SDM and transformation into *Escherichia coli* with ampicillin selection was carried out using the Q5 SDM kit (NEB) according to the manufacturer’s protocolusing primers 3, 4, 16 and 17 (Table S1). Verified plasmid minipreps of ΔE1E2FseI E1E2 cassettes were RE digested overnight with FseI (NEB, Fig. 1c) and then column purified.

Creating viable HCV chimeras requires the viral core and NS2 genes to be genotype-matched (Lindenbach *et al.*, 2005; Mateu *et al.*, 2008). A ΔCore-NS2AfeI cassette was therefore created to further test the strategy and facilitate the construction of chimeras with patient-derived Gt3 E1E2. J6/JFH-1(Lindenbach *et al.*, 2005) was screened in NEBcutter 2.0 for non-cutting RE sites and the component termini of AfeI were identified as naturally occurring at the desired chimeric junctions at the 5′ start of core (AGC) and the 3′ end of NS2 (GCT, Fig. 1h). Whilst the chosen parental HCV genome presented a fortuitous mutagenesis option, the RE digest mapping described above could simply be applied to select a novel non-cutting site, even the same FseI as used in the E1E2 region as this would be lost in the first DNA assembly cloning reaction. SDM primers 23 and 24 (Table S1) were then designed in NEB BaseChanger to remove the Core-NS2 sequence between the AGC and GCT junction points to create a complete AfeI RE site (AGCGCT). SDM and clone verification was performed (with insert screening primers 25 and 26, Table S1) to create a ΔCore-NS2 AfeI JFH-1 plasmid cassette (Fig. 1i). This process essentially removed the J6 component present in the parental chimera. The ΔCore-NS2 AfeI cassette was RE digested with AfeI (Thermo Fisher Scientific) and reverted to its parental wild-type using J6/JFH-1 core-NS2 amplified with PCR primers 27 and 28 (Table S1) by In-Fusion cloning (see below), to confirm unaltered phenotype. To create a novel HCV Gt3 ΔE1E2FseI cassette, a Core-NS2 Gt3 sequence (GenBank accession number GU814263.1, Gottwein *et al.*, 2007) was synthesized (Gene Strings, Invitrogen) already containing the above described ΔE1E2FseI modification and 15 bp terminal homology to the AfeI RE-digested ΔCore-NS2 AfeI cassette and cloned by In-Fusion (see below) into the ΔCore-NS2 AfeI JFH-1 plasmid cassette (Fig. 1j). This ΔE1E2FseI S52/JFH-1 cassette was verified using primers 25 and 26 and RE digested with FseI for In-Fusion cloning (Fig. 1c).

Forty-nine HCV E1E2 isolates (see Table S2) were derived from patients as previously described (Lavillette *et al.*, 2005; Owsianka *et al.*, 2005; Urbanowicz *et al.*, 2015). DNA clone sequences were aligned using clustalw, as implemented in the mega version 6 software (Tamura *et al.*, 2013), and complementary primers were designed to amplify the region between the aligned GG/CC junction points identified above (primers 5–13, 18–22 and 31–33, Table S1). Primers were also tagged with a 15 base region complementary to the appropriate end of the digested knockout cassette, ending in the GG dinucleotide motif (Fig. 1d). Plasmid template was amplified using the Q5 high-fidelity DNA polymerase (NEB, Fig. 1e) and PCR products inserted without purification into the cassette by In-Fusion cloning as per the manufacturer’s instructions (Fig. 1f).

In-Fusion reaction transformants were screened for presence of inserted DNA by colony PCR using cassette-specific primers (3 and 4, 16 and 17 and 29 and 30 for genotypes 1, 2 and 3 respectively, Table S1). Putative chimeric colony PCR product sequences were verified against parental ΔE1E2FseI E1E2 plasmid and patient-derived E1E2 sequences in mega6. Confirmed chimeric plasmids (Fig. 1g) were prepared and linearized overnight with XbaI (Thermo Fisher Scientific), column purified and used as a template to generate HCV RNA transcripts with a megascript T7 kit (Thermo Fisher Scientific). RNA transcripts were column purified using a QIAamp Viral RNA Mini Kit (Qiagen) and eluted in DEPC–treated water. Electroporation and cell culture was performed as previously described in duplicate (Lindenbach *et al.*, 2005).

To confirm no off-target deleterious PCR errors had occurred in the ΔE1E2FseI cassette constructs, preliminary wild-type revertant clones were created prior to the creation of chimeric clones and tested alongside the parental clone in the cell culture system. Parental wild-type E1E2 was reinserted into the cassettes by In-Fusion cloning with primers 5 and 7 (Gt 1, Table S1) and 19 and 22 (Gt2, Table S1) to confirm unaltered phenotype; alternatively full plasmid sequencing could be undertaken. Wild-type infectivity titres were demonstrated in cell culture for each of the wild-type reverted cassettes (data not shown).

The above strategy was initially applied to the previously described J6/JFH-1 parental chimera (Lindenbach *et al.*, 2005; Mateu *et al.*, 2008), generating a ΔE1E2 cassette to receive Gt2 E1E2s. Initially six patient-derived Gt2 E1E2s were selected based on their functionality in the pp assay (Lavillette *et al.*, 2005; Tarr *et al.*, 2011; Urbanowicz *et al.*, 2015). All of these isolates were able to replicate and produced infectious virus at all three sampled time points (24, 96 and 192 h post electroporation, Fig. 2b).

**Fig. 2.**
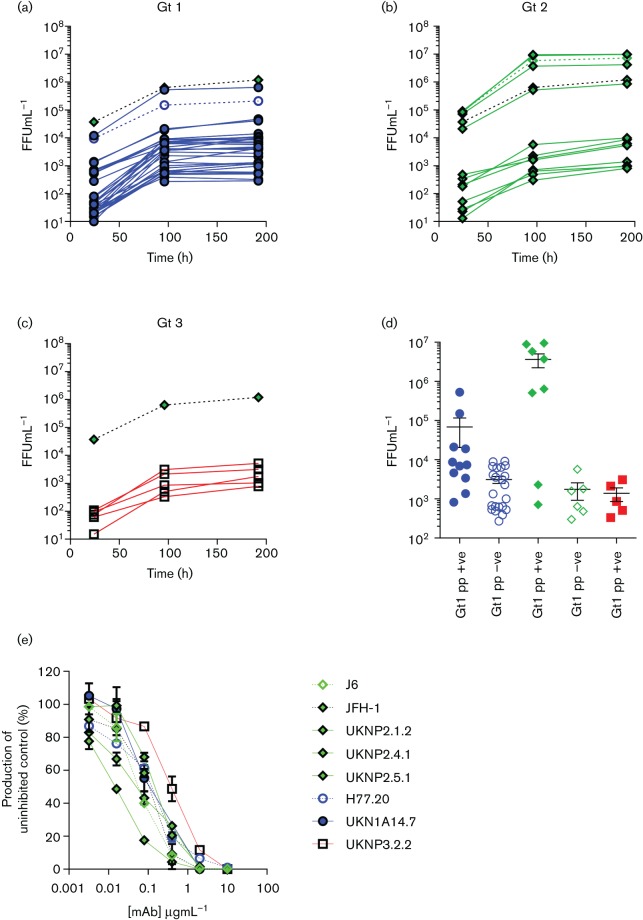
Forty-nine HCV E1E2 isolates show measurable infectivity in full-length chimeric HCV within 96 h of electroporation independent of function in a pseudoparticle assay. Focus forming units (FFU) were calculated from NS5a stained cells for each isolate at 24, 96 and 192 h time points. (a) Thirty-two genotype (Gt) 1 isolates, (b) 12 Gt2 isolates and (c) 5 Gt3 isolates are shown alongside the reference strains H77/JFH (Gt1, purple open circle), J6/JFH (Gt2, green open diamond) and JFH-1 (parental chimera; Gt2, green filled diamond). (d) FFU calculated for the 96 h time point for all 49 chimeras plotted against the isolate’s ability to produce infectious pseudoparticles above the limit of detection in a retroviral pseudotype assay (closed objects, termed pp +ve) or not (open objects, termed pp –ve). (e) Eight isolates with titres ranging from 1.2×10^3^ to 1.1×10^6^ FFU ml^−1^ were neutralized by an increasing concentration of anti-CD81 mAb. Mean±sem shown for each category.

Similarly ten Gt1 and five Gt3 E1E2 clones, which had previously been shown to confer entry in the HCVpp assay, were cloned into the H77/JFH-1 and S52/JFH-1 cassettes, respectively. Using this approach all of the clones were infectious (Fig. 2a, c). This confirms that infectious chimera construction relies on the presence of genotype matched core-NS2 genome segments, as has been described previously (Gottwein *et al.*, 2007; Lindenbach *et al.*, 2005; Mateu *et al.*, 2008; Pietschmann *et al.*, 2006).

The analysis was then extended to include E1E2 isolates that have been classed as non-functional in the pp assay (below the limit of detection, data not shown). Again, all 28 patient-derived E1E2 isolates (22 Gt1 and six Gt2) were both replication competent and produced infectious virus at the three sampled time points (Fig. 2a, b, c, d). To rule out the possibility of exosome transmission (Bukong *et al.*, 2014), we performed a neutralization assay with an anti-CD81 antibody (JS-81, BD Biosciences) on a select number of clones representative of high, medium and low virus titre, as previously described (Tarr *et al.*, 2013). Infectivity of all clones tested was completely blocked at 10 µg ml^−1^ (Fig. 2e).

The E1E2 clones used for chimera generation were all obtained from the UK. Despite their restricted geographical sampling they demonstrated a large degree of intra- genotype and subtype nucleotide diversity (Fig. 3). The intra-sample variability for the subtype 1a, 1b and 3a and the Gt2 clusters were 10.2, 11.6, 6.6 and 31.1 %, respectively, which is similar to that reported in previous phylogenetic studies (Brown *et al.*, 2007).

**Fig. 3.**
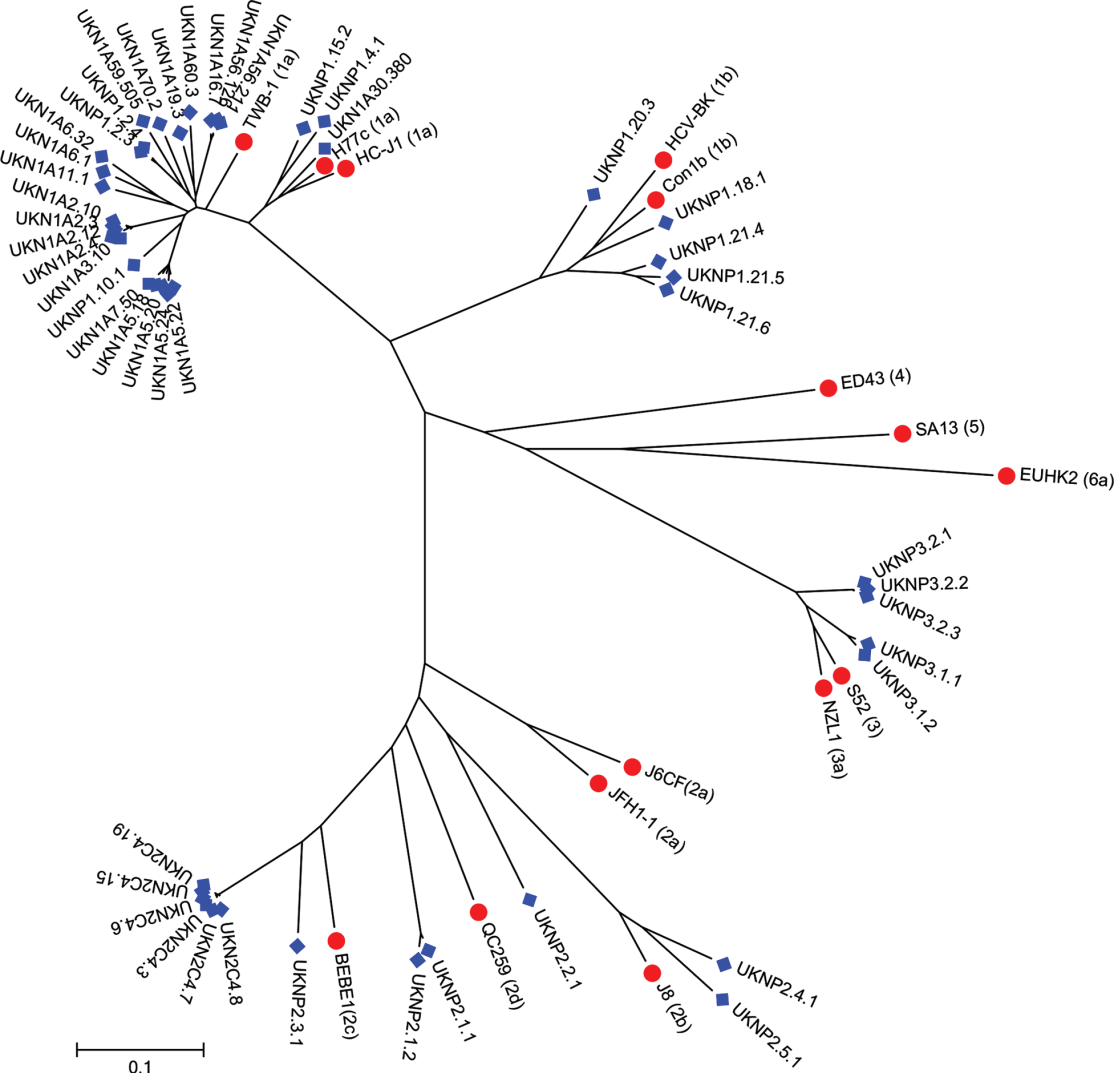
Maximum likelihood molecular phylogenetic analysis of HCV E1/E2 nucleotide sequences. The evolutionary history of the patient-isolated HCV E1/E2 genes cloned into chimeric HCVcc was inferred by using the maximum likelihood method based on the general time reversible model. The tree with the highest log likelihood is shown. Initial tree(s) for the heuristic search were obtained automatically by applying neighbour-joining and BioNJ algorithms to a matrix of pairwise distances estimated using the maximum composite likelihood approach, and then selecting the topology with superior log likelihood value. The tree is drawn to scale, with branch lengths measured in the number of substitutions per site. The analysis involved 49 E1/E2 gene sequences (blue diamond) derived from 25 individual patients, and 15 genotype reference nucleotide sequences (red circle). Codon positions included were 1st + 2nd + 3rd + non-coding. All positions containing gaps and missing data were eliminated. There were a total of 1649 positions in the final dataset. Evolutionary analyses were conducted in mega7 (Kumar *et al.*, 2016).

The inclusion of five Gt3 isolates is a leap forward and begins to reduce the paucity of isolates from this genotype. Previous studies have shown that patients harbour a high frequency of HCVpp entry-deficient E1E2 (Lavillette *et al.*, 2005). Crucially, the data presented here shows that the HCVpp system is not a good predictor of E1E2-mediated cell entry and this technique allows for a more diverse panel of E1E2s to be investigated. This technique has thus generated a panel of infectious HCV chimeras that is significantly larger than all the isolates produced to date and expanding it even further has now become readily achievable. Although the infectivity of some clones was low, it was still reproducible and at detection levels that would allow further phenotypic analysis, such as the neutralization assays presented in Fig. 2e. It is possible that further passage of the chimeras could lead to improved infectivity through the acquisition of adaptive mutations (Gottwein *et al.*, 2007; Mateu *et al.*, 2008), but care would be needed to ensure that these did not occur within the E1E2 coding region, or indeed other target regions, under study.

In summary, our method facilitates flexible and rapid creation of diverse, functional and seamless molecular chimeras for development and testing of novel therapeutics. This method can also be applied to personalized medicine by pre-testing patient-specific viruses for treatment sensitivity (Angus *et al.*, 2003; Imhof & Simmonds, 2011). Importantly, location of the cloning site is only limited by the need to identify an RE site not contained in the parental cassette backbone and, given that any RE digest overhang is removed during the cloning step, targeting to single nucleotide positions is possible. This gives a degree of flexibility and ease hitherto unheard of for a single-step chimeric reverse genetics procedure.

## Supplementary Data

62 Supplementary File 1

## Supplementary Data

107 Supplementary File 2

## References

[R1] Angus P. Vaughan R. Xiong S. Yang H. Delaney W. Gibbs C. Brosgart C. Colledge D. Edwards R.(2003). Resistance to adefovir dipivoxil therapy associated with the selection of a novel mutation in the HBV polymerase. Gastroenterology 125 292–297. 10.1016/S0016-5085(03)00939-9 12891527

[R2] Ball J. K. Tarr A. W. McKeating J. A.(2014). The past, present and future of neutralizing antibodies for hepatitis C virus. Antivir Res 105 100–111. 10.1016/j.antiviral.2014.02.013 24583033 PMC4034163

[R3] Brown R. J. Tarr A. W. McClure C. P. Juttla V. S. Tagiuri N. Irving W. L. Ball J. K.(2007). Cross-genotype characterization of genetic diversity and molecular adaptation in hepatitis C virus envelope glycoprotein genes. J Gen Virol 88 458–469. 10.1099/vir.0.82357-0 17251563

[R4] Bukong T. N. Momen-Heravi F. Kodys K. Bala S. Szabo G.(2014). Exosomes from hepatitis C infected patients transmit HCV infection and contain replication competent viral RNA in complex with Ago2-miR122-HSP90. PLoS Pathogens 10 . 10.1371/journal.ppat.1004424 PMC4183590 25275643

[R5] Burton D. R. Poignard P. Stanfield R. L. Wilson I. A.(2012). Broadly neutralizing antibodies present new prospects to counter highly antigenically diverse viruses. Science 337 183–186. 10.1126/science.1225416 22798606 PMC3600854

[R6] Chmielewska A. M. Naddeo M. Capone S. Ammendola V. Hu K. Meredith L. Verhoye L. Rychlowska M. Rappuoli R.(2014). Combined adenovirus vector and hepatitis C virus envelope protein prime-boost regimen elicits T cell and neutralizing antibody immune responses. J Virol 88 5502–5510. 10.1128/JVI.03574-13 24599994 PMC4019094

[R7] Das S. R. Hensley S. E. Ince W. L. Brooke C. B. Subba A. Delboy M. G. Russ G. Gibbs J. S. Bennink J. R. Yewdell J. W.(2013). Defining influenza A virus hemagglutinin antigenic drift by sequential monoclonal antibody selection. Cell Host Microbe 13 314–323. 10.1016/j.chom.2013.02.008 23498956 PMC3747226

[R28] deCamp A. Hraber P. Bailer R. T. Seaman M. S. Ochsenbauer C. Kappes J. Gottardo R. Edlefsen P. Self S.(2014). Global panel of HIV-1 Env reference strains for standardized assessments of vaccine-elicited neutralizing antibodies. J Virol 88 2489–2507. 10.1128/JVI.02853-13 24352443 PMC3958090

[R8] Dutta S. Dlugosz L. S. Drew D. R. Ge X. Ababacar D. Rovira Y. I Moch J. K. Shi M. Long C. A.(2013). Overcoming antigenic diversity by enhancing the immunogenicity of conserved epitopes on the malaria vaccine candidate apical membrane antigen-1. PLoS Pathogens 9 . 10.1371/journal.ppat.1003840 PMC3873463 24385910

[R9] Edmonds T. G. Ding H. Yuan X. Wei Q. Smith K. S. Conway J. A. Wieczorek L. Brown B. Polonis V.(2010). Replication competent molecular clones of HIV-1 expressing Renilla luciferase facilitate the analysis of antibody inhibition in PBMC. Virology 408 1–13. 10.1016/j.virol.2010.08.028 20863545 PMC2993081

[R10] Gottwein J. M. Scheel T. K. Hoegh A. M. Lademann J. B. Eugen-Olsen J. Lisby G. Bukh J.(2007). Robust hepatitis C genotype 3a cell culture releasing adapted intergenotypic 3a/2a (S52/JFH1) viruses. Gastroenterology 133 1614–1626. 10.1053/j.gastro.2007.08.005 17983807

[R11] Imhof I. Simmonds P.(2010). Development of an intergenotypic hepatitis C virus (HCV) cell culture method to assess antiviral susceptibilities and resistance development of HCV NS3 protease genes from HCV genotypes 1 to 6. J Virol 84 4597–4610. 10.1128/JVI.02698-09 20164226 PMC2863784

[R12] Imhof I. Simmonds P.(2011). Genotype differences in susceptibility and resistance development of hepatitis C virus to protease inhibitors telaprevir (VX-950) and danoprevir (ITMN-191). Hepatology 53 1090–1099. 10.1002/hep.24172 21480315

[R13] Irwin C. R. Farmer A. Willer D. O. Evans D. H.(2012). In-fusion® cloning with vaccinia virus DNA polymerase. Methods Mol Biol 890 23–35. 10.1007/978-1-61779-876-4_2 22688759

[R14] Keck Z. Wang W. Wang Y. Lau P. Carlsen T. H. Prentoe J. Xia J. Patel A. H. Bukh J. Foung S. K.(2013). Cooperativity in virus neutralization by human monoclonal antibodies to two adjacent regions located at the amino terminus of hepatitis C virus E2 glycoprotein. J Virol 87 37–51. 10.1128/JVI.01941-12 23097455 PMC3536422

[R15] Kumar S. Stecher G. Tamura K.(2016). MEGA7: molecular evolutionary genetics analysis version 7.0 for bigger datasets. Mol Biol Evol 33 1870–1874. 10.1093/molbev/msw054 27004904 PMC8210823

[R16] Lavillette D. Tarr A. W. Voisset C. Donot P. Bartosch B. Bain C. Patel A. H. Dubuisson J. Ball J. K. Cosset F. L.(2005). Characterization of host-range and cell entry properties of the major genotypes and subtypes of hepatitis C virus. Hepatology 41 265–274. 10.1002/hep.20542 15660396

[R17] Lindenbach B. D. Evans M. J. Syder A. J. Wölk B. Tellinghuisen T. L. Liu C. C. Maruyama T. Hynes R. O. Burton D. R.(2005). Complete replication of hepatitis C virus in cell culture. Science 309 623–626. 10.1126/science.1114016 15947137

[R18] Mateu G. Donis R. O. Wakita T. Bukh J. Grakoui A.(2008). Intragenotypic JFH1 based recombinant hepatitis C virus produces high levels of infectious particles but causes increased cell death. Virology 376 397–407. 10.1016/j.virol.2008.03.027 18455749 PMC2492671

[R19] Owsianka A. Tarr A. W. Juttla V. S. Lavillette D. Bartosch B. Cosset F. L. Ball J. K. Patel A. H.(2005). Monoclonal antibody AP33 defines a broadly neutralizing epitope on the hepatitis C virus E2 envelope glycoprotein. J Virol 79 11095–11104. 10.1128/JVI.79.17.11095-11104.2005 16103160 PMC1193588

[R20] Pietschmann T. Kaul A. Koutsoudakis G. Shavinskaya A. Kallis S. Steinmann E. Abid K. Negro F. Dreux M.(2006). Construction and characterization of infectious intragenotypic and intergenotypic hepatitis C virus chimeras. Proc Natl Acad Sci U S A 103 7408–7413. 10.1073/pnas.0504877103 16651538 PMC1455439

[R21] Reyes-del Valle J. de la Fuente C. Turner M. A. Springfeld C. Apte-Sengupta S. Frenzke M. E. Forest A. Whidby J. Marcotrigiano J.(2012). Broadly neutralizing immune responses against hepatitis C virus induced by vectored measles viruses and a recombinant envelope protein booster. J Virol 86 11558–11566. 10.1128/JVI.01776-12 22896607 PMC3486281

[R22] Steinmann E. Doerrbecker J. Friesland M. Riebesehl N. Ginkel C. Hillung J. Gentzsch J. Lauber C. Brown R.(2013). Characterization of hepatitis C virus intra- and intergenotypic chimeras reveals a role of the glycoproteins in virus envelopment. J Virol 87 13297–13306. 10.1128/JVI.01708-13 24089562 PMC3838269

[R23] Tamura K. Stecher G. Peterson D. Filipski A. Kumar S.(2013). MEGA6: molecular evolutionary genetics analysis version 6.0. Mol Biol Evol 30 2725–2729. 10.1093/molbev/mst197 24132122 PMC3840312

[R24] Tarr A. W. Urbanowicz R. A. Hamed M. R. Albecka A. McClure C. P. Brown R. J. Irving W. L. Dubuisson J. Ball J. K.(2011). Hepatitis C patient-derived glycoproteins exhibit marked differences in susceptibility to serum neutralizing antibodies: genetic subtype defines antigenic but not neutralization serotype. J Virol 85 4246–4257. 10.1128/JVI.01332-10 21325403 PMC3126256

[R25] Tarr A. W. Lafaye P. Meredith L. Damier-Piolle L. Urbanowicz R. A. Meola A. Jestin J. L. Brown R. J. McKeating J. A.(2013). An alpaca nanobody inhibits hepatitis C virus entry and cell-to-cell transmission. Hepatology 58 932–939. 10.1002/hep.26430 23553604

[R26] Urbanowicz R. A. McClure C. P. Brown R. J. Tsoleridis T. Persson M. A. Krey T. Irving W. L. Ball J. K. Tarr A. W.(2015). A diverse panel of hepatitis C virus glycoproteins for use in vaccine research reveals extremes of monoclonal antibody neutralization resistance. J Virol 90 3288–3301. 10.1128/JVI.02700-15 26699643 PMC4794667

[R27] You S. Stump D. D. Branch A. D. Rice C. M.(2004). A cis-acting replication element in the sequence encoding the NS5B RNA-dependent RNA polymerase is required for hepatitis C virus RNA replication. J Virol 78 1352–1366. 10.1128/JVI.78.3.1352-1366.2004 14722290 PMC321395

