# Supplementary material for: Flexible and rapid construction of viral chimeras applied to hepatitis C virus
Source: J Gen Virol. 2016 Sep;97(9):2187–93. doi: 10.1099/jgv.0.000530 (PMC5042125; doi:10.1099/jgv.0.000530)
Supplement: Supplementary File 1 [file jgv-97-2187-s001.pdf]

Primer Number	Primer name	Purpose	Target region	Sequence (5' to 3')
1	H77_ΔFse_SDMf	SDM	H77 E1E2	GGCCAAGCGGAGGCGGCTTT
2	H77_ΔFse_SDMr	SDM	H77 core	GGCCAGGAAGGTTCCCTGTTGC
3	H77_E1E2_SCRf	E1E2 Screening	H77 core	GCGCAATTTGGGTAAGGTCA
4	H77_E1E2_SCRr	E1E2 Screening	H77 NS2	ACCCTTCAGATACCACGCAA
5	G1E1E2>H77Fa	E1E2 In-Fusion	H77 / 1a E1E2	AGGGAACCTTCCTGGTTGCTCTTTCTCTATC
6	G1E1E2>H77Fb	E1E2 In-Fusion	Gt1 E1E2	AGGGAACCTTCCTGGTTGCTCTTTCTCTATCTTCCTTC
7	G1E1E2>H77Ra	E1E2 In-Fusion	Gt1 E1E2	GCCGCCTCCGCTTGGGATATGAGTAACATCATCCA
8	G1E1E2>H77Rb	E1E2 In-Fusion	Gt1 E1E2	GCCGCCTCCGCTTGGGCTATGAGTAACACCATCCA
9	G1E1E2>H77Rc	E1E2 In-Fusion	Gt1 E1E2	GCCGCCTCCGCTTGGGCTACCAGCAGCATCATCCA
10	G1E1E2>H77Rd	E1E2 In-Fusion	Gt1 E1E2	GCCGCCTCCGCTTGGGCTATCAGCAGCATCATCCA
11	G1E1E2>H77Re	E1E2 In-Fusion	Gt1 E1E2	GCCGCCTCCGCTTGGGCTATGAGTAGCATCATCCA
12	G1E1E2>H77Rf	E1E2 In-Fusion	Gt1 E1E2	GCCGCCTCCGCTTGGGATATGAGTAGCATCATCCA
13	G1E1E2>H77Rg	E1E2 In-Fusion	Gt1 E1E2	GCCGCCTCCGCTTGGGCTATGAGTAACATCATCCA
14	J6_ΔFse_SDMf	SDM	J6 E1E2	CCGGCCGAAGCAGCACTA
15	J6_ΔFse_SDMr	SDM	J6 core	CCGGGTAAGTTCCCTGTTGC
16	J6_E1E2_SCRf	Screening	J6 core	GCTTTGCCGACCTCATG
17	J6_E1E2_SCRr	Screening	J6 NS2	GTACCAAGCAGCCACGAAAA
18	G2E1E2>J6Fa	E1E2 In-Fusion	Gt2 E1E2	AGGGAACTTACCCGGTTGCTCTTTTTCTATC
19	G2E1E2>J6Fb	E1E2 In-Fusion	J6 E1E2	AGGGAACTTACCCGGTTGCTCCTTTTCTATC
20	G2E1E2>J6Fc	E1E2 In-Fusion	JFH-1 E1E2	AGGGAACTTACCCGGTTTCCCCTTTTCTATC
21	G2E1E2>J6Ra	E1E2 In-Fusion	Gt2 E1E2	CTAGTGCTGCTTCGGCTTGGCCCA
22	G2E1E2>J6Rb	E1E2 In-Fusion	J6 / JFH-1 E1E2	CTAGTGCTGCTTCGGCCTGGCCCA
23	J6_ΔAfeI_SDMf	SDM	J6 Core	GCTCCCATCACTGCTTATGCCCAGCAAACAC
24	J6_ΔAfeI_SDMr	SDM	J6 NS2	GCTCATGGTGCACGGTCT
25	JFH_C-NS2_SCRf	Screening	JFH-1 5'UTR	ACTCTATGCCCGGCCATTT
26	JFH_C-NS2_SCRr	Screening	JFH-1 NS3	GGCCTGTTCTGTCCTGTCA
27	J6_C-NS2>JFHf	CoreNS2 In-Fusion	J6 Core-NS2	CCGTGCACCATGAGCACAAATCCTAAACCTCAAAGAA
28	J6_C-NS2>JFHr	CoreNS2 In-Fusion	J6 Core-NS2	AGCAGTGATGGGAGCGAGAAGACTCCACCCCTT
29	G3_E1E2_SCRf	Screening	Gt3 E1E2	CGTAGGAGGCGTCGCAAG
30	G3_E1E2_SCRr	Screening	Gt3 E1E2	AGCCAATACCATGTGTCCCA
31	G3E1E2>G3Fa	E1E2 In-Fusion	Gt3 E1E2	AGGGAACTTGCCCGGTTGCTCCTTTTCTATCTTCC
32	G3E1E2>G3Fb	E1E2 In-Fusion	Gt3 E1E2	AGGGAACTTGCCCGGTTGCTCTTTCTCTATCTTC
33	G3E1E2>G3R	E1E2 In-Fusion	Gt3 E1E2	AGCATCAGCCAAAGGGCAACGCACACGC

Supplementary table 1: Primers for PCR amplification. Primer numbers, names, target gene, breadth of target and sequence are detailed. HCV genotype is abbreviated to 'Gt' and 15 base 5' homology tags are indicated in grey.
