# Supplementary material for: Flexible and rapid construction of viral chimeras applied to hepatitis C virus
Source: J Gen Virol. 2016 Sep;97(9):2187–93. doi: 10.1099/jgv.0.000530 (PMC5042125; doi:10.1099/jgv.0.000530)
Supplement: Supplementary File 2 [file jgv-97-2187-s002.pdf]

Isolate	Accession number
UKN1A2.3	KX389139
UKN1A2.4	KX389140
UKN1A2.10	KX389141
UKN1A2.12	KX389142
UKN1A3.10	KX389143
UKN1A5.18	KX389144
UKN1A5.20	KX389145
UKN1A5.22	KX389146
UKN1A5.24	KX389147
UKN1A6.1	KX389148
UKN1A6.32	KX389149
UKN1A7.50	KX389150
UKN1A11.1	KX389151
UKN1A14.7	KU213640
UKN1A16.7	KX389152
UKN1A30.380	KX389153
UKN1A56.126	KX389154
UKN1A56.211	KX389155
UKN1A59.505	KX389156
UKN1A19.3	KX389157
UKN1A60.3	KX389158
UKN1A70.2	KX389159
UKN2C4.3	KX389160
UKN2C4.6	KX389161
UKN2C4.7	KX389162
UKN2C4.8	KX389163
UKN2C4.15	KX389164
UKN2C4.19	KX389165
UKNP1.2.3	KU285154
UKNP1.2.4	KU285155
UKNP1.4.1	KU285161
UKNP1.10.1	KU285172
UKNP1.15.2	KU285186
UKNP1.18.1	KU285192
UKNP1.20.3	KU285198
UKNP1.21.4	KU285191
UKNP1.21.5	KU285205
UKNP1.21.6	KU285206
UKNP2.1.1	KU285209
UKNP2.1.2	KU285210
UKNP2.2.1	KU285211
UKNP2.3.1	KU285212
UKNP2.4.1	KU285213
UKNP2.5.1	KU285214
UKNP3.1.1	KU285216
UKNP3.1.2	KU285215
UKNP3.2.1	KU285218
UKNP3.2.2	KU285219
UKNP3.2.3	KU285217
